# Case of Spinal Irritation, Menstruation during Utero-gestation, Frequent Abortion, &c.

**Published:** 1840-03-28

**Authors:** A. Hester

**Affiliations:** New Orleans


### MEDICAL EXAMINER.

DEVOTED TO MEDICINE, SURGERY, AND THE COLLATERAL SCIENCES.

No. 13.] PHILADELPHIA, SATURDAY, MARCH 28, 1840. [Vol. III.

### *Case of Spinal Irritation, Menstruation during Utero-gestation, frequent Abortion, &c.* By A. Hester, M. D.

On the 14th of April, 1839, I saw Mad. W. for the first time, æt. 25, of a nervo-sanguineous temperament, considerable "embonpoint," complexion shaded, muscles soft, skin remarkably delicate. She was now, according to her calculation, in the latter part of the third month of utero-gestation; had been pregnant five times previously; three abortions between the third and fourth months, and was threatened with the fourth, for which my advice was sought. I found her with skin hot, face flushed, eyes animated, pulse frequent, nausea and occasional vomiting, with irregular lancinating, and bearing down pains in the hypogastric regions.

Venesection, rest, cooling, gentle laxatives, "lac cum aqua calcis pro nauseam," were prescribed. Failing to relieve the pains by these simple measures, I prescribed the salts of morphine, enemata of laudanum, &c.; warm baths, fomentations, and other soothing applications. These had the desired effect.

*April 15th.*—Intolerance of light; extreme mobility of nervous system. Inherits an idiosyncrasy, which renders the use of opium and its preparations objectionable; pain under left mamma. When attempting to examine the pulse, she shrank from the slightest touch, complaining of great tenderness in the tegumentary surface of both arms, and over the thorax.

I was immediately struck with the present symptoms in this case, as being analogous, or precisely similar, to those in a female patient presented to the medical class by Dr. S. Jackson, at Blockley Hospital, with spinal irritation. I seized this symptom, and requested to examine the spine, which was found extremely painful on the slightest pressure, from the fourth cervical to the last lumbar vertebræ, inclusive.

A fact very impressive in this case was, an increase of pain under the left breast, on pressing the fingers along that portion of the spine which gave off nerves to the upper part of the chest. Scarified cups to the painful points in the course of the vertebral column followed by a subsidence of almost all pain about the chest; pulse much reduced in force and frequency, soft and regular; skin warm and quite moist; declared herself better in all respects; confessed she had been affected, during her previous pregnancies, with precisely similar symptoms, but stated, the spine did not attract the attention of her physician.

Such a complication led me to a more minute and thorough investigation into the history of the case. Teale, in his interesting little work on Neuralgia, observes, that spinal irritation may persist, in some instances, for five or six years, according to habit, constitution, &c. This must have been a case of chronic or subacute irritation or inflammation of the cord or its membranes, or both, rendered acute or aggravated from causes constitutional, or the presence of fœtus in utero reacting upon an iritable cerebro-spinal axis. If the above suggestions derive any support from the laws of pathology, it is interesting to remark, that, as has been repeatedly stated, though phthisis, with all its direful train of evils, may be apparently suspended during gestation, yet in the subject before us, not only from my own careful observations, but likewise from the accurate and lucid statements of this intelligent lady, the spinal affection was unquestionably more painful and manifest during the period of utero-gestation. Hence it follows, that while disease in one organ may be masked or arrested in its course, in another it may be renewed, developed, and rendered more obvious through the mysterious influence of sympathy with a third organ, when called upon to assume the important office of reproduction. How far this pathologic law may apply, I am unable to determine.

Another striking feature in the case under review, and which I have embraced in the synopsis, was the non-interruption of the menstrual discharge from the time of conception up to the seventh month of gestation.

Such departures from the ordinary laws of the female economy, are acknowledged to be rare.

This was observed to have occurred in all the five pregnancies previous to the present, at least until abortion was induced in three, and up to the seventh month in the two others. I am not inclined to believe both were deceived in this matter. The patient protested that the discharges recurred regularly, that the product was in every essential similar in quality, as well as the same, or nearly so, in quantity, save, however, that she was less indisposed while it lasted in the unimpregnated state of the uterus, than during gestation. I was also informed that this lady's mother suffered no interruption of the catamenial flow during her pregnancies.

Such was the accuracy of this patient's calculations, as to the time the flow would return, that the day was often specified when I might expect to be summoned to her bed side, since their irruption was accompanied with general constitutional disturbances, which threatened abortion. But cups down the spine, revulsives and anodynes, &c., in a few days usually subdued all alarming symptoms. This patient was also much troubled with obstinate pruritus vulvæ. The children born by this lady were of delicate frames, and feeble constitutions, and usually died at the end of twelve months with some complaint of the gastro-enteric surfaces. At the expiration of the nine months, this lady was delivered of a healthy and perfectly formed child.

*New Orleans*, March 5, 1840.

